# Integrating Anaerobic Digestion and Hydrothermal Liquefaction for Maximum Energy Recovery: A Techno-Economic Assessment and Comparison

**DOI:** 10.1021/acs.energyfuels.5c06644

**Published:** 2026-07-30

**Authors:** Christian Klüpfel, Hendrik Etzold, Ahmad Kordi, Mintu Monica Thomas, Benjamin Herklotz, Patrick Biller

**Affiliations:** † 130422 Deutsches Biomasseforschungszentrum gemeinnützige GmbH, Torgauer Straße 116, Leipzig 04347, Germany; ‡ Department of Biological and Chemical Engineering, 1006 Aarhus University, Hangøvej 2, Aarhus N DK-8200, Denmark

## Abstract

The increasing global energy demand combined with the growing global population requires technologies to sustainably provide energy. Arguably the most established scheme to convert wet waste biomass to energy is anaerobic digestion (AD) to produce biogas. The side product, digestate, can pose both ecological and economic challenges for the process. Hydrothermal liquefaction (HTL) can be employed to treat and valorize the digestate to produce biocrude oil. In this study, the integration of AD and HTL for sewage sludge, straw/manure, and biogenic household waste is techno-economically assessed and compared to the HTL of the respective undigested biomass. Higher biocrude yield, and thus energy recovery, was found when using the undigested biomass, yet the overall energy recovery is higher by up to 2-fold when combining AD and HTL. The experimental data was used to inform a process model using Aspen Plus software. A preliminary assessment at an input mass stream of 10 kt a^–1^ reveals minimum biocrude selling prices (MBSP) of biocrude oil between 4.6 and 16.4 € kg^–1^ depending on feedstock and processing temperature. The economically most viable scenario for both AD + HTL and HTL alone was straw/manure due to higher biocrude yield and higher earnings through biomethane sales. These two cases were scaled up to a simulation of a full-scale plant of 250 kt a^–1^. The cost analysis reveals that consumption-linked costs contribute >73% of total annual cost. MBSP is 1.6 € kg^–1^ for AD + HTL and 1.8 € kg^–1^ for HTL. The sensitivity analysis highlights production quantity and AP treatment as determining price drivers, while energy and labor costs are secondary. Integrating AD and HTL can increase the overall energy yield while enhancing the techno-economic validity of the process.

## Graphical abstract

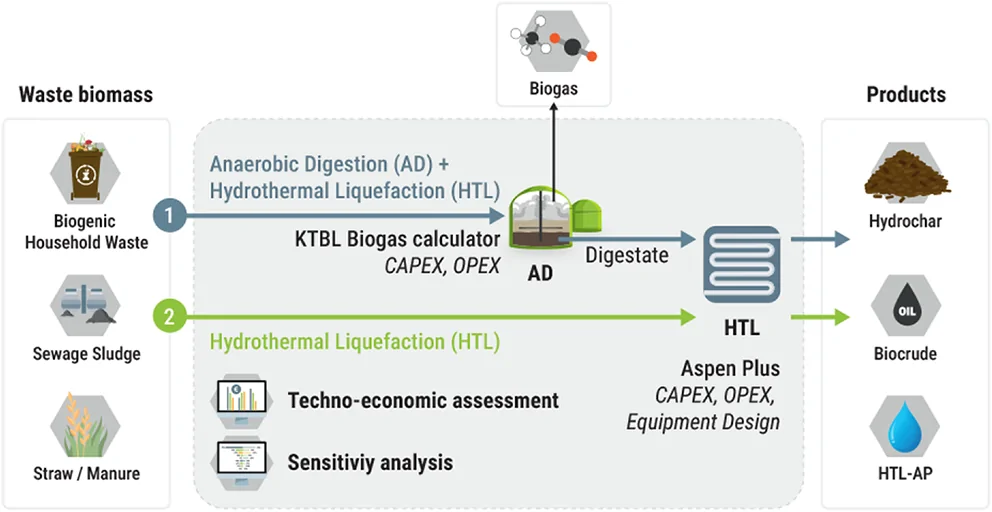

## 1 Introduction

The scalability of advanced biofuels is fundamentally constrained by the availability and sustainability of low-risk biomass feedstock. First-generation, crop-derived biofuels face trade-offs in terms of land-use change risks and unfavorable lifecycle emissions in many cases,^1^ which has driven policy and industry toward residues, wastes, and advanced feedstocks as the preferred near-term route for SAF deployment. An analysis by the International Council on Clean Transportation (ICCT) indicates that SAF blending targets defined under the ReFuelEU Aviation regulation can be met using waste- and residue-based biomass, such as agricultural and urban residues, available in the EU.^2^

Hydrothermal liquefaction (HTL) has been well demonstrated as a technically viable route to convert wet, heterogeneous biogenic residues, including sewage sludge, straw, manure, and the organic fraction of municipal waste, at temperatures around 250–400 °C and reaction times of 10–60 min.^3^ The main product, biocrude oil, is a fuel precursor that can be upgraded to be used in aviation and shipping.^4^ Digestate from anaerobic digestion (AD) is widely considered a less favorable feedstock due to low biocrude yields, high ash, and low total solids (TS) content. Digestate management in biogas plants raises both ecological and economic concerns. Direct land application of digestate can cause a nutrient surplus in the soil, causing and affecting nitrate-vulnerable zones. Policies limiting the amount that can be applied to agricultural fields entail the need for storage and transportation, which can heavily affect the economics of AD.^5^

Previous techno-economic assessments of biocrude and SAF production from digestate and other biomass have indicated the viability of the hydrothermal route. Hoffmann et al. (2013)^6^ performed an Aspen study with a conceptual design of combining AD and HTL, including the upgrading of the biocrude. The HTL unit is fed with the digestate from the fermenter, while biogas is used for (1) generation of electricity (CHP), (2) hydrogen production, and (3) heat generation (gas boiler). Biocrude produced is upgraded in the upgrading unit, with hydrogen produced from biogas. Energy efficiency of the plant is 84% in the best scenario. Economic assessment was not part of this study. Okoro et al.^7^ performed an experimental study of digestate treatment via HTL followed by a TEA. Their calculation suggests that HTL can cut costs by 85% compared to established treatment methods, yet the authors did not consider the value of HTL products. Del Alamo et al. (2022)^8^ ran an extensive TEA of sewage sludge HTL, calculating the cost of biocrude production between 10–40 € GJ^–1^ and 20–60 € GJ^–1^ for the upgraded jet fuel depending on the scale of the plant. Deuber et al. (2023)^9^ assessed the production of sustainable aviation fuel (SAF) from lignocellulosic residues in Brazil using HTL and showed similar price ranges as the aforementioned authors, depending on the solvent used. Hussain and Anastasakis (2025)^10^ investigated the production cost of biocrude from sewage sludge. Their base case of roughly 15 kt a^–1^ at a dry matter content of 20% results in a biocrude price of 1.4 € kg^–1^. Sensitivity analysis reveals biocrude yield, labor cost, and sludge dry matter content as the highest risk parameters. To the best of the authors’ knowledge, no studies considering both AD and HTL in the TEA exist. The aim of this study is to techno-economically assess and compare the two resulting biorefinery schemes: the HTL of digestate, i.e., performing AD and HTL (termed *AD + HTL*), and the HTL of undigested biomass (termed *HTL*). Three typical biomass scenarios, sewage sludge, agricultural waste, and biogenic household waste, were selected. Experimental and plant operator data were used to build an Aspen Plus process model and provide a preliminary economic assessment at a base case of 10 kt a^–1^. For each of the two biorefinery scenarios investigated, one was selected for a more detailed assessment at a scale of 250 kt a^–1^ to elucidate price drivers and risk factors through a rigorous sensitivity analysis.

## 2 Materials and Methods

### 2.1 Materials

Sewage sludge (SS) and digested sewage sludge (DSS), biogenic waste (BW) and digested biogenic waste (DBW), as well as straw/manure (SM) and straw/manure digestate (SMD), are selected to represent three of the most important cases of anaerobic digestion. SS and DSS were acquired from a large local municipal wastewater treatment plant located in Leipzig, Germany, before and after digestion for 24 d. The BW is a mixture of green cuttings and biogenic household waste, which is anaerobically digested in thermophilic mode with a water content of 72–74% for 10–15 d. SM is a mixture of 18.25% straw and 81.25% manure that was anaerobically digested at the German Biomass Research Center (Deutsches Biomasseforschungszentrum gemeinnützige GmbH, DBFZ) in continuous operation with a retention time of 18 d. All biomass was dried until constant mass at 105 °C and ground in a cutting mill equipped with a 1 mm sieve to ensure homogenization. Along with the biomass, plant operator data regarding biogas yield and composition was obtained.

### 2.2 Experimental Procedure

HTL experiments were performed in 20 mL custom-built batch reactors consisting of piping (OD = 20 mm, wall = 2 mm) and fittings. For each experiment, 2 g of biomass were loaded into the reactors along with 10 g of DI water and 0.06 g KOH as a base catalyst. HTL was performed at 300–350 °C for 20 min, and the experiments were run in duplicates. The methodology is described in detail in a previous paper.^11^

The yield of each product phase was calculated based on the mass of initial feedstock:

1 $$Y_{\mathrm{Phase}}=\frac{M_{\mathrm{Phase}}}{M_{\mathrm{Feed}}}$$

wherein Phase denotes the respective product phase biocrude, hydrochar, or gas. The amount of water-soluble products was calculated by difference. Energy recovery (ER) specifies the amount of energy contained in the feedstock that is transferred to a product phase represented by the ratio of their respective higher heating values (HHV):

2 $$\mathrm{ER}=Y_{\mathrm{Phase}}\cdot \frac{HHV_{Phase}}{HHV_{Feed}}$$

### 2.3 Analytical Methods

Biomass was analyzed for crude fat, crude fiber, protein content, acid detergent fiber, neutral detergent fiber, and nitrogen-free extracts (NfE) using methods described elsewhere.^12^ Carbohydrate content was calculated by summing up NfE and raw fiber. Elemental analysis (C, H, N, S) of biomass, biocrude oils, and hydrochars was conducted according to DIN EN 15104 using an elemental analyzer (vario MACRO CUBE, Elementar Analysensysteme GmbH, Langenselbold, Germany). Ash content of digestates and hydrochars was determined gravimetrically by incineration at 550 °C according to DIN EN 14775. Oxygen content was then calculated by difference. The HHV of digestates, biocrude oils, and hydrochars was calculated based on their elemental composition according to the correlation by Channiwala and Parikh (2002).^13^ A comprehensive overview of feedstock properties is given in Table 1.

**Table 1:** Feedstock Analysis

	Sewage sludge (SS)	Digested sewage sludge (DSS)	Straw/manure (SM)	Straw/manure digestate (SMD)	Biogenic waste (BW)	Digested biogenic waste (DBW)
Ash [% DM]	29.51	42.34	11.72	18.25	27.42	35.46
N [% DM]	6.28	4.15	1.28	1.71	1.73	2.18
C [% DM]	38.54	31.53	47.95	43.80	40.21	36.47
H [% DM]	5.10	4.48	5.38	5.44	4.73	4.35
S [% DM]	0.86	1.43	0.27	0.37	0.18	0.32
O [% DM]	19.71	16.07	33.40	30.43	25.73	21.22
HHV [MJ/kg]	16.79	13.81	19.38	18.20	16.36	14.95
Al [g/kg]	6610	7490	315	381	4990	7930
Ca [g/kg]	22900	30100	9940	15900	20600	33300
Fe [g/kg]	62500	24500	563	2730	5590	7380
Mg [g/kg DM]	4670	6410	4280	6510	2520	4530
Na [g/kg DM]	2030	3940	844	1790	2160	4520
P [g/kg DM]	34700	22300	2960	4990	3360	5700
K [g/kg]	6920	9510	28100	44400	8220	16400
Protein [% DM]	28.36	36.60	7.53	12.43	9.89	16.19
Crude fat [% DM]	7.81	7.06	6.60	4.09	4.85	3.68
Crude fiber [% DM]	7.91	8.91	37.29	26.05	27.61	25.48
Lignin [% DM]	19.06	21.58	9.22	15.14	12.94	26.16
Nitrogen-free extracts [% DM]	26.86	8.69	37.30	41.39	30.00	19.17
Total carbohydrates [% DM]	34.77	17.60	74.59	67.44	57.61	44.65

### 2.4 Process Description and Simulation

The process *AD + HTL* consists of a digestion of the original biomass followed by hydrothermal liquefaction of the digestate, while *HTL* stands for hydrothermal liquefaction of the original biomass. For the simulation of the hydrothermal process, Aspen Plus (v14, Aspen Technology, Inc., Bedford, MA, USA) is used. A high-pressure pump is used to feed the biomass into the heat exchanger, which harnesses the product stream heat to heat the biomass stream. The reaction takes place in an RYIELD reactor, which resembles the yields of the product phases found in the lab-scale experiments. Solid separation is achieved using a hydrocyclone. After the heat exchanger, a cooler is used to cool down the mixture of gas, aqueous phase, and biocrude. A final pressure release valve reduces the pressure to atmospheric, and the product mixture is separated in a settling tank. The flow sheet is depicted in Figure 1. In Aspen Plus, the Soave–Redlich–Kwong cubic equation of state is used for all thermodynamic properties. Biomass, digestate, and hydrochar are modeled as nonconventional solids using the property models HCOALGEN for enthalpy and DCOALIGT for density. Required proximate, ultimate, and sulfonate analyses are given. Biocrude oil and aqueous phase are simulated as a mixture of 13 compounds most prevalent in GC–MS analysis^11^ to fit elemental and TC/TN analysis, respectively.

**Figure 1:**
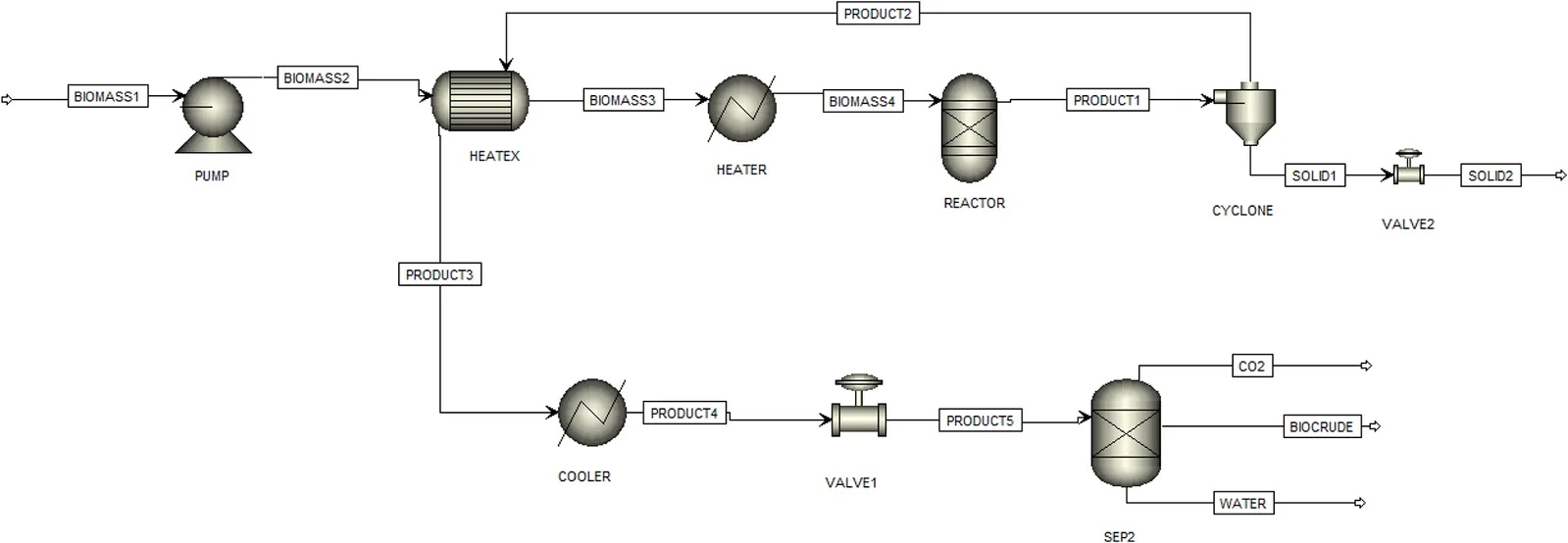
Flow sheet of the Aspen model.

The heat exchanger is simulated as a shell and tube design with the hot fluid situated in the shell. Design parameters were set using the Aspen exchanger design and rating tool. Specifications can be found in the Supporting Information. According to Tubular Exchangers Manufacturers Association (TEMA) standards, the head is designed as B (bonnet bolted), the shell as E (one pass), and the rear head as M (bonnet). For the simulation and design, the outer diameter of the tube is fixed to 0.75 in., and the heat transfer efficiency is set to 85%. The reactor in the Aspen simulation is merely simulated as a YIELD reactor for mass and energy balancing without further technical details. For cost calculations, a tubular reactor with a diameter of 0.1 m and a length of 1210 m is designed to ensure turbulent flow. The hydrocyclone was simulated at an efficiency of 0.8.

For the anaerobic digestion plant, the economic calculation tool by the *Kuratorium für Technik und Bauwesen in der Landwirtschaft e.V.* (KTBL) is used. The German organization provides data and software tools for the agricultural industry, including a profitability calculator for biogas plants. This calculator is a specialized tool using data from practice and industry to provide reliable economic data. Biogas is upgraded to biomethane using a membrane process and subsequently fed into the gas grid. All relevant plant equipment, i.e., pumps, conveyors, fermenter, post-digester, measurement and control, membrane unit, and biogas feed-in plant, are accounted for. Biomethane revenue is calculated depending on the scenario with long-term sales prices from 2024, which are 0.11 € kWh^–1^ for SS and BW (residues) and 0.19 € kWh^–1^ for SM, ascertained by the German Energy Agency.^14^

### 2.5 Cost Estimation

Cost estimation and economic analysis in this study are based on equipment cost estimation, as suggested by Buchner (2020)^15^ for a technology readiness level (TRL) of 4–7. The Aspen Plus Economic Analyzer (APEA) is used for a preliminary economic assessment to compare the six biomasses at three temperatures (300, 325, and 350 °C) for a total of 18 base cases at 10 kt a^–1^ (wet) of input biomass with TS = 20%. The two economically most viable cases, one each for *AD + HTL* and *HTL*, were then scaled up to a full-scale plant of 250 kt a^–1^. Equipment design parameters were used to obtain a comparative cost assessment for the heat exchanger, the pump, and the reactor using the Dutch association of cost engineers (DACE) price booklet,^16^ methods by Peters et al. (2004),^17^ and data from Matches.^18^ The Chemical Engineering Plant Cost Index (CEPCI) was used to adapt all prices for the year 2024. Detailed economic and sensitivity analysis is then performed using the in-house biorefinery economic assessment tool (BEAT). The tool is mainly based on the VDI guideline 6025,^19^ with adjustments in particular to the methodologies from Peters et al. (2004)^17^ and Turton.^20^ CAPEX was allocated to individual years by means of annuity with the capital recovery factor (CRF):

3 $$CRF=\frac{{\left(1+i\right)}^{n}\times i}{{\left(1+i\right)}^{n}-1}$$

where *i*, the interest rate, is assumed to be 0.05, and *n*, the lifetime of the plant, is assumed to be 10 a. This results in a CRF = 0.1295, which is factored with CAPEX to calculate the annuity. A sensitivity analysis is used to determine the influence of various factors on the specific production costs for the scaled-up plant at 250 kt a^–1^. Relevant input variables for the calculation are selected and then varied. The selected parameter is gradually lowered or increased, starting from the base value assumed in the calculation. The course of the target variable is observed. Increasing sensitivity results in higher validity of the parameter because factors with a high influence on the target variable entail a high risk to the reliability of the results.

#### 2.5.1 CAPEX and Equipment Cost

Equipment prices for the HTL plant were first obtained from the APEA. Design specifications were then used to obtain comparative estimations from methods by Peters et al. (2004),^17^ the DACE price booklet, and Matches to arrive at reliable and consolidated cost estimations. Direct (e.g., instrumentation, piping, electrical systems) and indirect (e.g., construction, engineering, legal, contingencies) costs were calculated from equipment cost using factors to determine the estimated overall cost. Direct costs were split into inside battery limits (ISBL) and outside battery limits (OSBL). An overview of all factors and base assumptions used in the detailed assessment is summarized in Table 2.

**Table 2:** Cost Base Factors Used for the Detailed Assessment

Capital costs		Factor of equipment cost
Direct costs		
Equipment cost		100%
Delivery		10%
ISBL	Equipment installation	39%
	Instrumentation & control	26%
	Piping	31%
	Electrical systems	10%
OSBL	Buildings (including services)	29%
	Yard improvements	12%
	Service facilities	55%
Indirect costs		
	Engineering and supervision	32%
	Construction expenses	34%
	Legal expenses	4%
	Contractor’s fee	19%
	Contingency	37%
Base factors		
	Base year	2024
	Assessment period (in years)	10
	Interest rate	5%
	Annuity factor a	0.1295

#### 2.5.2 OPEX

Operational expenditure (OPEX) included biomass cost, labor cost, side stream treatment, energy cost, and consumption-linked cost. If applicable, typical biomass market prices in Germany were assumed. Raw and auxiliary materials were assumed at market prices of 110 € t^–1^, 5 € t^–1^, and 646 € t^–1^ for straw, manure, and KOH, respectively. Wastewater treatment was assumed to be done off-site and calculated using TOC and TN data from product analysis with prices from Leipziger Stadtwerke, which accounted for 0.001 € m^–3^ for each mg_TOC_ L^–1^ and 0.006 € m^–3^ for each mg_TNB_ L^–1^. Electricity was assumed at 0.15 € kWh^–1^ and natural gas at 0.07 € kWh^–1^ with data from Eurostat for nonhousehold consumers for the second half of 2024 in the corresponding consumption bands.^21,22^

## 3 Results and Discussion

### 3.1 Mass and Energy Balance

The mass and energy yield resulting from lab-scale batch experiments, represented by mass yield and energy recovery, is compiled in Table 3. A comprehensive overview of the mass and energy balance for all 18 cases is compiled in the Supporting Information. The two main energy products, biogas/biomethane and biocrude, are considered. The methane yield differs widely between the three scenarios, from 13.0% for SS to 3.05% for BW, which can be accounted to the higher lignin content of BW. Generally, 1.2–1.5-fold higher biocrude mass yields were found with the undigested material compared to the digestate. SM yields the highest amount of biocrude, followed by SS and BW; this is consistent for the digestate. All biocrudes are of usual quality, around 28–34 MJ/kg, which tends to increase with temperature and is unaffected by type of biomass. The ER_Biocrude_ increases with temperature up to 39.28%. The overall energy yield of the appropriate process ER_total_ increases by 1.5–1.8- fold and 1.3–1.5-fold, respectively, when combining *AD + HTL*. Thus, up to 58.10% of the energy of the original biomass can be energetically exploited when combining the two processes, as compared to 43.04% and 39.28% when only AD or HTL is applied, highlighting an interesting synergy between the two processes. For BW, HTL seems to be the energetically more favorable path.

**Table 3:** Mass and Energy Yield of Biocrude and Biomethane

Feedstock	Process	T_HTL_ [°C]	Y_Methane_ [Ma. %]	ER_Biogas_ [%]	Y_Biocrude_ [Ma. %]	HHV [MJ/kg]	ER_Biocrude_ [%]	ER_total_ [%]
Sewage sludge	AD + HTL	300	13.00	43.04	13.02	30.28	28.64	57.21
		325			12.81	32.55	30.27	58.02
		350			12.58	33.30	30.43	58.10
	HTL	300	-	-	15.60	34.21	31.83	31.83
		325			17.09	31.69	32.31	32.31
		350			19.19	34.31	39.28	39.28
Straw/manure	AD + HTL	300	10.09	28.96	20.50	28.70	32.36	48.59
		325			19.68	30.69	33.23	49.11
		350			19.08	33.07	34.71	50.01
	HTL	300	-	-	21.07	29.68	32.35	32.35
		325			23.53	30.52	37.15	37.15
		350			22.59	31.15	36.40	36.40
Biogenic waste	AD + HTL	300	3.05	10.38	9.75	32.64	21.33	27.79
		325			9.30	33.49	20.90	27.43
		350			10.52	29.58	20.87	27.41
	HTL	300	-	-	14.66	30.31	27.26	27.26
		325			15.04	32.31	29.81	29.81
		350			15.96	32.59	31.91	31.91

### 3.2 Influence of Temperature and Biomass on Price

The experimental data was used to set up a model in Aspen Plus. In total, 18 models were created for the base case of 10 kt a^–1^, representing the three HTL processing temperatures across three digested (*AD + HTL*) and three undigested (*HTL*) biomasses. Feedstock TS content was assumed to be a constant 20% for all biomass to ensure an even comparison. Built-in APEA was used to obtain CAPEX and OPEX. The results of the preliminary economic assessment are summarized in Table 4. CAPEX for the HTL plant was between 2,800 and 3,400 k€, mainly influenced by higher processing temperature and thus higher pressure rating for the equipment. This cost is in good agreement with the HTL technology of Circlia Nordic ApS, which is marketed at 3,500 k€^23^ for a wet waste input stream capacity of 25 kt a^–1^.^24^ Major cost contributors in equipment cost were the heat exchanger (around 130–140 k€), reactor (120–180 k€), and pump (260–280 k€), equating to roughly 85% of equipment cost, which aligns well with previous findings.^10,25^ A full account of the cost, as well as heat exchanger and hydrocyclone design, is given in the Supporting Information. Estimation of CAPEX for the biogas plant obtained through the KTBL biogas calculator is very similar for the three biomasses in the range of 2,700–2,800 k€. The cost of the membrane process amounts to 1,300 k€, thus making the largest contribution. Annuity is calculated by factoring CRF (3) with CAPEX. Biogas plant operating cost is mostly negligible in the range of 55–75 k€, while HTL operating cost from Aspen Plus makes up the lion’s share in the range of 1,200–1,600 k€. Process water treatment adds annual costs of 350–800 k€. This large deviation is due to higher TN in process water from SS and DSS, causing significantly higher cost than SM and BW. Heating of the HTL process costs 55–85 k€ depending on process temperature. Biomethane sales are calculated with the amount of biomethane produced and the respective sales price for each biomass. These can vary heavily, on the one hand by the quantity produced, but also by the price achieved, as laid out in Section 2.4. Among the feedstocks, straw/manure generally produces the cheapest biocrude with a minimum biocrude selling price (MBSP) ranging from 7.44 to 8.33 € kg^–1^ for *AD + HTL* and 4.32 to 4.73 € kg^–1^ for *HTL*. In all cases, the MBSP from *AD+HTL* is higher than from *HTL* due to lower biocrude yield. The economically most viable cases for each process are straw/manure at 300 °C for *AD + HTL* and straw/manure at 325 °C for *HTL.* These two cases are selected for scale-up and a more detailed cost analysis.

**Table 4:** Preliminary Economic Assessment

Feedstock	Process	T_HTL_ [°C]	Biomethane sales [€/y]	CAPEX [€]	Annuity [€/y]	OPEX [€/y]	Total deficit [€/y]	MBSP [€/kg]	HHV [MJ/kg]	MBSP [€/GJ]
Sewage sludge	AD + HTL	300	440,809	5,836,469	755,849	2,056,261	2,371,302	15.12	30.28	499.22
		325		6,055,397	784,202	2,067,976	2,411,369	15.63	32.55	480.26
		350		6,300,806	815,983	2,117,378	2,492,552	16.44	33.30	493.86
	HTL	300	-	2,986,283	386,737	1,989,506	2,376,244	7.62	34.21	222.70
		325		3,202,527	414,742	2,001,267	2,416,009	7.07	31.69	223.10
		350		3,378,630	437,548	2,037,811	2,475,359	6.45	34.31	188.02
Straw/manure	AD + HTL	300	578,460	5,845,669	757,041	1,753,849	1,932,429	7.44	28.70	259.12
		325		6,058,755	784,636	1,765,555	1,971,731	7.90	30.69	257.50
		350		6,143,630	795,628	1,798,158	2,015,326	8.33	33.07	251.98
	HTL	300	-	3,013,356	390,243	1,569,034	1,959,278	4.73	34.21	138.25
		325		3,211,599	415,917	1,581,232	1,997,148	4.32	31.69	136.27
		350		3,285,136	425,440	1,614,208	2,039,648	4.59	34.31	133.86
Biogenic waste	AD + HTL	300	103,394	5,732,567	742,394	1,610,367	2,249,366	12.94	32.64	396.37
		325		5,895,584	763,505	1,621,991	2,282,102	13.75	33.49	410.47
		350		6,091,225	788,841	1,658,134	2,343,581	12.49	29.58	422.09
	HTL	300	-	2,962,696	383,683	1,559,259	1,942,942	6.63	30.31	218.63
		325		3,162,314	409,534	1,573,668	1,983,202	6.59	32.31	204.05
		350		3,453,896	447,295	1,604,024	2,051,319	6.43	32.59	197.19

### 3.3 Estimation and Comparison of Equipment Prices

The cases yielding the lowest cost biocrude by mass for each biorefinery scenario, i.e., straw/manure *HTL* at 325 °C and straw/manure *AD+HTL* at 300 °C, were scaled up in Aspen Plus to a full-scale plant with a capacity of 250 kt a^–1^ of input biomass, and equipment prices were calculated using APEA. The three most expensive units, heat exchanger, pump, and reactor, are subjected to a price comparison using the characteristic equipment design parameters from Aspen Plus. This is heat transfer area, volumetric flow, and volume for the heat exchanger, pump, and reactor, respectively. The results are displayed in Table 5. For the heat exchanger, DACE, Matche, and Peters estimate a similar cost of 54–80 k€, while Aspen projects roughly 317 k€, i.e., a 4-fold price. The pump is estimated by Matche to cost 170–182 k€, which is in the middle of APEA and Peters. For the reactor, the APEA estimation of 302–314 k€ equals roughly 5-fold the Peters estimation of 57 k€. Aspen Plus cost estimation is widely used and trusted in research and industry for preliminary cost acquisition. In this case, for all considered equipment, comparatively high prices are found by APEA, which deviate from the other estimation databases many times over. HTL of waste biomass is a novel process with little to no TRL 9 implementations. Generally, most plants range between TRL 7 and 8. The mass balance data, on which this model is based, was acquired at a small scale (20 mL) in the lab. As such, we think a conservative cost estimation approach is sensible. The overall cost degression from demo-scale (10 kt a^–1^) to full-scale (250 kt a^–1^) in APEA follows an exponent of 0.85, which is in the expected range of [0.6; 0.9] and suggests some degree of viability. As such, the APEA estimation is selected as a *worst-case* scenario for this assessment.

**Table 5:** Cost Estimation Comparison of Heat Exchanger, Pump, and Reactor

		Characteristic parameter			Cost estimation [€]			
Equipment	Process	A [m^2^]	V̇ [m^3^/h]	V_R_ [m^3^]	APEA	DACE	Matche	Peters
Heat exchanger	AD + HTL	65.50	-	-	288,379	80,419	55,510	73,697
	HTL	62.68	-	-	288,015	79,079	54,236	70,750
Pump	AD + HTL	-	28.12	-	302,757	-	169,969	62,201
	HTL	-	29.80	-	338,065	-	181,669	79,196
Reactor	AD + HTL	-	-	5.69	302,211	-	-	56,783
	HTL	-	-	5.87	313,859	-	-	56,784

### 3.4 Cost and Sensitivity Analysis

In our preliminary assessment, straw/manure showed the best economic performance for both *AD + HTL* and *HTL*. An in-depth analysis is performed at an input stream of 250 kt a^–1^ (wet) or 136 dry tons per day to show cost drivers and structures in more detail. The cost structure of the *AD + HTL* plant and the *HTL* plant are shown in Figures 2 and 3, respectively. Both plants are dominated by consumption-linked cost, which makes up >70% of annual cost. In the case of *AD + HTL,* this includes 6.5 M€ for raw and auxiliary materials (straw, manure, KOH), 6.5 M€ for AP treatment, 3.6 M€ for electricity, and 0.4 M€ for natural gas for heating. The HTL plant requires 343 kW for pumping and 1701 kW for heating the reactor. Annual capital is calculated based on equipment cost estimation, which is 1.3 M€ for HTL, both for *AD + HTL* and *HTL*; thus, the increase in processing temperature from 300 to 325 °C shows no significant increase in equipment cost. Direct (e.g., installation, instrumentation, buildings) and indirect (engineering, contingency, construction) costs, calculated through the factor method, give a CAPEX of 5.0 M€. The overall factor between equipment cost and total cost is 4.38. The AD plant has an estimated CAPEX of 18.7 M€; main cost contributors are 10 main and post fermenters at 10.5 M€ and the membrane unit for biomethane separation for 4.5 M€. This gives a fixed capital investment for the *AD + HTL* process of 21.8 M€, which results in a CRF of 2.5 M€ a^–1^ or 410 € t^–1^ of biocrude. In the case of the *HTL* process, this is 65 € t^–1^. This means that only 4% and 11%, respectively, of the total annual cost is made up of capital cost, despite the assumption of the highest cost estimation for the scale-up. Operating cost includes the labor cost, estimated via plant capacity according to Peters et al.,^17^ which contributes 1.4 M€ and 2 M€, respectively, for *HTL* and *AD + HTL*. Other costs, including insurance and uncertainties, are negligible with 120 and 33 € t^–1^. The *AD + HTL* process additionally yields revenue from biomethane sales, namely 13,500 k€ a^–1^ or 2,226 € t^–1^. The resulting MBSP amounts to 1,601 € t^–1^ (equivalent to 53 € GJ^–1^ and 6.46 € per gasoline gallon equivalent (GGE)) for *AD + HTL* and 1,761 € t^–1^ (equivalent to 56 € GJ^–1^ and 6.73 € GGE^–1^) for *HTL*. Studies into the techno-economics of coliquefaction of straw and manure are pending. A recent study by Del Alamo et al. (2022)^8^ estimates the MBSP of a unit this size processing sewage sludge to be 16 € GJ^–1^. The simulated process ran at a higher dry matter content of 23% and yielded more biocrude (29 Ma. %) at a higher HHV of 35.2 MJ/kg, all of which contribute to a roughly 70% lower MBSP. Deuber et al. (2023)^9^ estimate an MFSP of upgraded biocrude from sugarcane straw between 20 and 52 € GJ^–1^ at a plant capacity of 1124 dry metric tons per day, which is roughly 10 times larger than ours, and a biocrude yield of 40.9%. The most recent report regarding the TEA of wet waste biomass by PNNL (2022) estimated MBSP from sewage sludge to 2.40 $ GGE^–1^ at a dry matter input stream of 110 dry tons per day. Hussain and Anastasakis (2025)^10^ concluded an MBSP from sewage sludge of 0.9–1.8 € kg^–1^ depending on plant size, which resembles our range. Generally, this places our findings in the upper end of the interval in terms of MBSP, in part due to lower biocrude yield. All articles considered undigested biomass, which seems to improve HTL economic performance. No other studies investigating the TEA of HTL of digestate were found. The results of the sensitivity analysis are displayed in Figures 4 and 5 for *AD + HTL* and *HTL*. The depiction is in the format of a tornado diagram to show the influence of selected cost contributors on production cost (i.e., MBSP) to illustrate its possible variance. The diagram is sorted by maximum amplitude of cost variation, while the corresponding interval was selected individually for each parameter. The most significant parameter, production quantity, is shown from [+50; −20]%, equating to a biocrude yield distribution of [16.4; 30.8] Ma. %, which reflects current research findings.^5,26^ This parameter can mainly be influenced by biocrude yield and dry matter content of the biomass. Feedstock quality (affected by seasonal and regional variety) and process optimization can be good levers to increase biocrude yield and significantly decrease production cost. For an increase of 50%, MBSP can be decreased by 80% to 325 € t^–1^. AP treatment is yet again found to be among the most crucial economic barriers for the HTL technology. By mass, around 80% of the effluent stream is wastewater; its treatment in this case is estimated at 30 € t^–1^. Thus, the levers show a range of 15–45 € t^–1^, which accounts for the national and regional variance of cost for heavily polluted wastewater. Cutting AP treatment cost by half, i.e., to 15 € t^–1^, can help reduce MBSP to 1071 € t^–1^. Novel and established processes, such as wet oxidation and anaerobic digestion, could help alleviate this burden, yet detailed economic studies are pending. Ceragioli et al. (2025)^27^ have shown the potential of an autothermal integrated HTL and wet oxidation unit, significantly decreasing energy footprint and increasing energy return on investment. Investment cost for the combined *AD + HTL* process is 3.8 times higher than for the *HTL* plant; as such, the associated price lever is greater. In both cases, capital investment is found to have the third highest cost amplitude. For this analysis, the highest cost estimation from the comparison (see Section 3.3) was used, yet a higher investment cost should always be considered when investigating immature technology. Doubling investment cost can increase MBSP 1.40- and 1.16-fold, respectively. Energy cost, estimated through Aspen Plus and KTBL calculator, is identified as more relevant in the *AD + HTL* case, as process energy requirement increases from 2,365 kW for *HTL* to 3,582 kW for the combined process. Price data for electricity and natural gas were taken from Eurostat in times of high energy prices for industry. Future cost development is difficult to predict; these data only represent a snapshot in time. Cost variations associated with operating and raw materials, labor, and a given period can be viewed as negligible.

**Figure 2:**
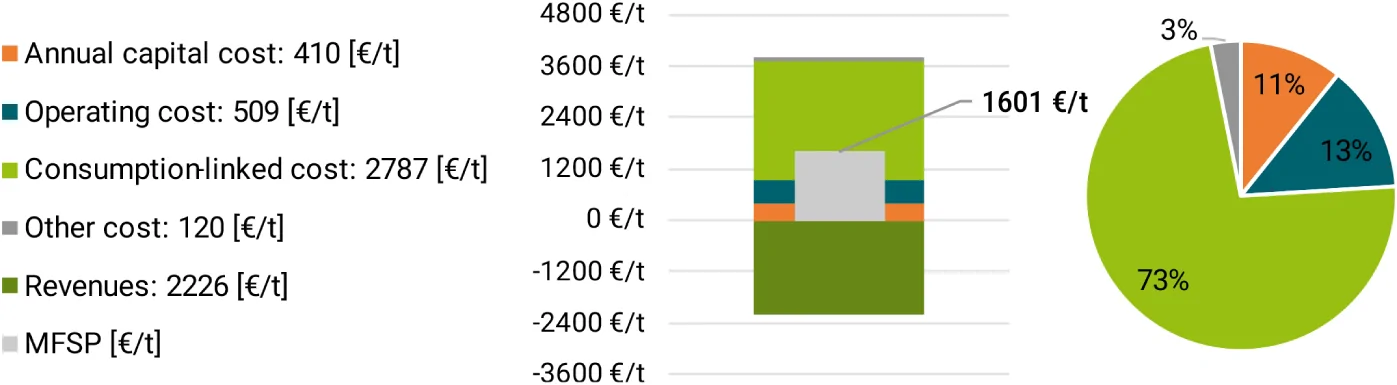
Cost structure of the AD + HTL plant.

**Figure 3:**
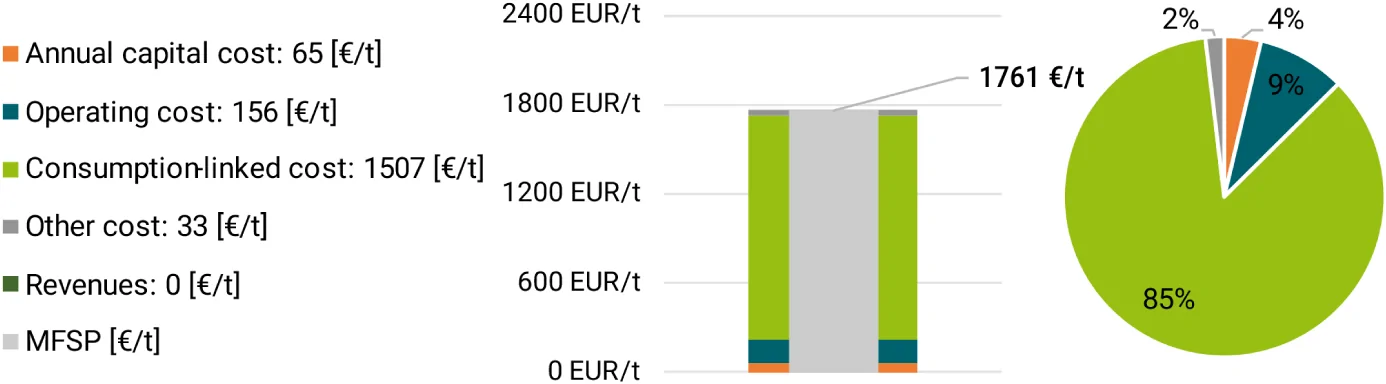
Cost structure of the HTL plant.

**Figure 4:**
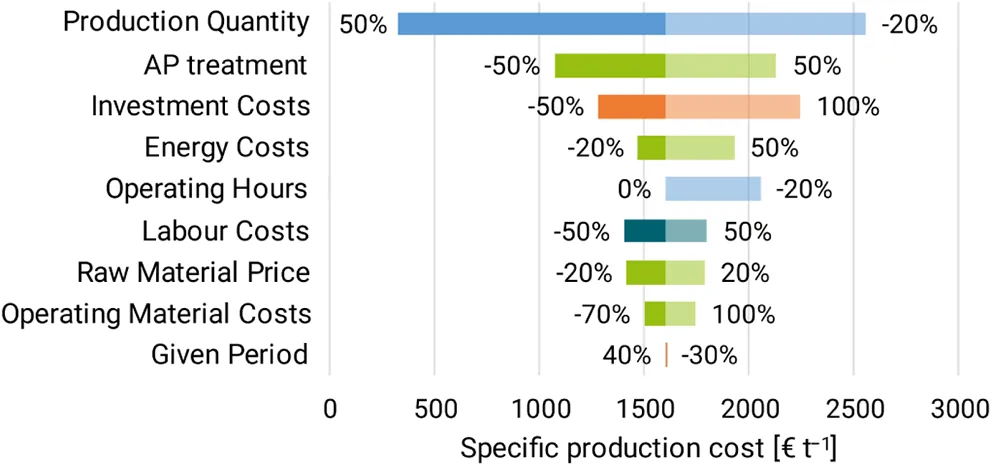
Tornado diagram for the AD + HTL biorefinery.

**Figure 5:**
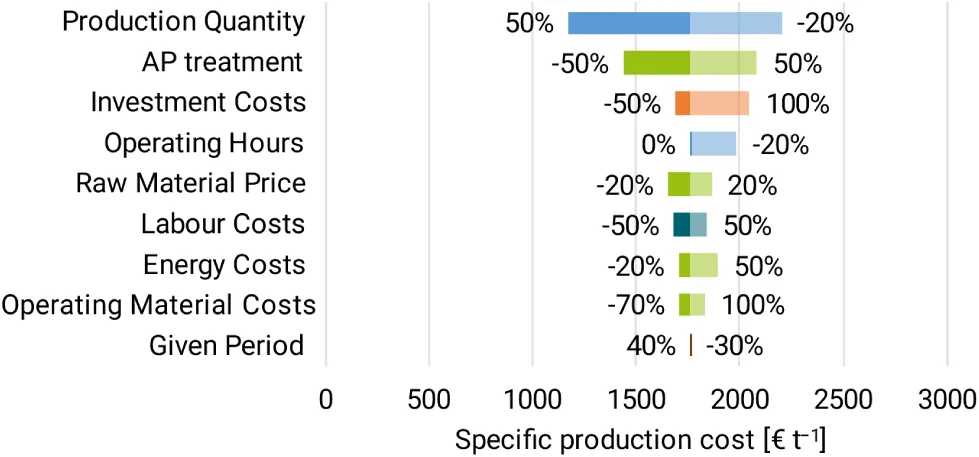
Tornado diagram for the HTL plant.

### 3.5 Limitations of This Study and Implications for Future Research

This study is subject to several sources of uncertainty that may affect the robustness of the TEA. Among other factors, the biocrude yield is strongly influenced by seasonal and regional variations in feedstock composition, which may affect the process performance and overall conversion efficiency. For *AD +* HTL, digestate quality is further influenced by the operation of the AD process. In addition, AP treatment costs are highly site-dependent and can vary substantially with the location of the plant and the organic load of the nearby wastewater treatment facilities. Consequently, the reported MBSPs should be interpreted only as indicative values, with greater emphasis placed on the outcomes of the sensitivity analysis rather than on the absolute figures.

The analysis identifies the biocrude production quantity, AP treatment cost, and capital investment as the most influential parameters shaping the overall production cost for both investigated cases. Further research should focus on optimizing these aspects, particularly the development of cost-effective AP treatment strategies. Although investment cost represents a smaller share of the total cost structure, variations in capital expenditure can significantly affect the MBSP and should, therefore, not be neglected in future assessments.

Digestate is often considered an unsuitable HTL feedstock because of its high ash and low organic content. This study demonstrates that fully integrating AD into the process framework enhances overall mass, energy, and economic performance. Biocrude can be produced at a lower cost, while total energy recovery from the biomass can increase by up to 2-fold.

The extensive deployment of biogas plants in Europe, coupled with persistent economic and environmental challenges in digestate management, reinforces the potential of the integrated *AD + HTL* biorefinery concept as a viable pathway toward more sustainable and efficient bioresource utilization.

## 4 Conclusion

The investigated biorefining scheme of AD + HTL can make sense energetically depending on the biomass used as feedstock, facilitating an up to 2-fold increase of the energetic exploitation of the biomass. The preliminary assessment at 10 kt a^–1^ highlights the agricultural case (i.e., straw/manure) as the economically most viable. After scale-up, a more detailed assessment and sensitivity analysis reveal production quantity, AP treatment, and investment cost as the main price drivers. The minimum biocrude selling price is estimated to be 53 and 56 € GJ^–1^ for *AD + HTL* and *HTL*, respectively. Combining AD and HTL can allow for a marginally lower fuel selling price while significantly increasing the overall energy yield from waste biomass.

## Supplementary Material



## Notes

Mass and energy balance, equipment and installed cost from Aspen Plus, heat exchanger, and hydrocyclone design specifications (PDF)
