# Supplementary material for: Integrating Anaerobic Digestion and Hydrothermal Liquefaction for Maximum Energy Recovery: A Techno-Economic Assessment and Comparison
Source: Energy Fuels. 2026 Jul 30;40(32):17627–35. doi: 10.1021/acs.energyfuels.5c06644 (PMC13488269; doi:10.1021/acs.energyfuels.5c06644)
Supplement: Supplementary file 1 [file ef5c06644_si_001.pdf]

Supporting Information

Integrating anaerobic digestion and hydrothermal liquefaction for maximum energy recovery: a techno-economic assessment and comparison

Christian Klüpfel[1*], Hendrik Etzold[1], Ahmad Kordi[1], Mintu Monica Thomas[1], Benjamin Herklotz[1], Patrick Biller[2]

[1]Deutsches Biomasseforschungszentrum gemeinnützige GmbH, Torgauer Straße 116, 04347 Leipzig, Germany

[2]Department of Biological and Chemical Engineering, Aarhus University, Hangøvej 2, DK-8200, Aarhus N, Denmark

[*]Corresponding author

Table S1 Mass and energy balance of anaerobic digestion at 10 kt a^{-1}

Biomass	FM Feed [t/y]	TS [%]	Water Feed [t/y]	TS Feed [t/y]	HHV Feed [MJ/kg]	Educt Energy stream [GJ/y]	Gas Yield [m³/Kg Ts]	Methane Yield [m³/Kg Ts]	Gas production [t/y]	Methane production [t/y]	Methane Yield [%]	Energy production [GJ/y]	ER [%]	Energy stream digestate [t/y]	CO2-production [t/y]	Methane production [m3/y]	Methane production [kWh/y]
Sewage sludge	10000	20	8000	2000	16.76	33,520	0.33	0.20	795.37	259.94	13.00	14426	43.04	19,094	535.43	395,641	4,007,351
Straw/manure	10000	20	8034	1966	19.33	38,003	0.28	0.15	698.38	198.32	10.09	11007	28.96	26,996	500.07	301,854	3,057,401
Biogenic waste	10000	20	8000	2000	16.30	32,600	0.09	0.05	215.65	60.97	3.05	3384	10.38	29,216	154.68	92,800	939,948

Table S2 Mass and energy balance of HTL of digested and undigested biomass at 10 kt a⁻¹

Biomass	FM Feed [t/y]	TS [%]	Water Feed [t/y]	TS Feed [t/y]	Educt Energy stream [GJ/y]	T [°C]	Yield Biocrude [%]	HHV [MJ/kg]	Biocrude mass [t/y]	Energy production [GJ/y]	ER [%]	ERtotal [%]	Energy production total [GJ/y]	Yield Gas [%]	Gas mass [t/y]	Yield AP [%]	AP mass [t/y]	Yield Char [%]	Char mass [t/y]	HHV Char [MJ/kg]	ER Char [%]	specific energy production [MJ/Kg TS]	specific energy production total [MJ/Kg TS]
Digested Sewage sludge	9,205	13.09	8,000	1,205	16,588	300	13.02	30.28	156.87	4,750	28.64	57.21	19,176	5.25	63.24	29.02	349.58	52.71	634.96	8.26	0.32	3.94	9.59
						325	12.81	32.55	154.26	5,021	30.27	58.02	19,447	7.23	87.09	29.54	355.85	50.42	607.37	7.47	0.27	4.17	9.72
						350	12.58	33.30	151.58	5,047	30.43	58.10	19,474	9.24	111.31	30.05	361.99	48.13	579.79	6.72	0.24	4.19	9.74
Straw/ manure digestate	9,302	13.63	8,034	1,268	23,045	300	20.50	28.70	259.86	7,458	32.36	48.59	18,464	13.07	165.68	39.39	499.31	27.04	342.76	20.08	0.30	5.88	9.39
						325	19.68	30.69	249.51	7,657	33.23	49.11	18,664	14.22	180.26	41.40	524.79	24.70	313.10	19.25	0.26	6.04	9.49
						350	19.08	33.07	241.82	7,998	34.71	50.01	19,005	15.93	201.93	44.24	560.79	21.94	278.12	18.35	0.22	6.31	9.67
Digested Biogenic waste	9,784	18.24	8,000	1,784	26,605	300	9.75	32.64	173.89	5,675	21.33	27.79	9,059	10.75	191.82	30.58	545.66	48.93	873.08	12.92	0.42	3.18	4.53
						325	9.30	33.49	166.03	5,560	20.90	27.43	8,944	10.48	187.00	34.64	618.10	45.58	813.31	14.02	0.43	3.12	4.47
						350	10.52	29.58	187.70	5,552	20.87	27.41	8,936	11.22	200.20	33.55	598.65	44.72	797.96	13.81	0.41	3.11	4.47
Sewage Sludge	10,000	20	8,000	2,000	33,520	300	15.60	34.21	311.91	10,670	31.83	-	-	8.22	164.44	35.27	705.47	40.91	818.20	9.35	22.83	0.02	-
						325	17.09	31.69	341.78	10,829	32.31	-	-	5.49	109.80	39.15	783.07	38.27	765.40	9.07	20.71	0.02	-
						350	19.19	34.31	383.75	13,166	39.28	-	-	5.98	119.60	38.68	773.53	36.16	723.20	4.94	10.66	0.02	-
Straw/ Manure	10,000	20	8,034	1,966	38,003	300	21.07	29.68	414.28	12,295	32.35	-	-	15.44	303.55	43.46	854.41	20.03	393.79	22.84	23.67	6.25	-
						325	23.53	30.52	462.54	14,117	37.15	-	-	14.95	293.92	43.50	855.29	18.02	354.27	19.94	18.59	7.18	-
						350	22.59	31.15	444.15	13,835	36.40	-	-	15.48	304.34	45.59	896.24	16.06	315.74	21.26	17.67	7.04	-
Biogenic waste	10,000	20	8,000	2,000	32,600	300	14.66	30.31	293.17	8,887	27.26	-	-	11.97	239.40	37.25	745.03	36.12	722.40	13.42	29.74	4.98	-
						325	15.04	32.31	300.77	9,719	29.81	-	-	12.97	259.40	39.90	798.00	32.1	642.00	13.60	26.78	5.45	-
						350	15.96	32.59	319.22	10,403	31.91	-	-	15.46	309.20	36.48	729.60	32.1	642.00	10.07	19.84	5.83	-

Table S3 Equipment and installed cost from Aspen Plus for the 10 kt a^{-1} case

**Biomass**	**DSS**	**DSS**	**DSS**	**SS**	**SS**	**SS**	**SM**	**SM**	**SM**	**DSM**	**DSM**	**DSM**	**DBW**	**DBW**	**DBW**	**BW**	**BW**	**BW**
**Temperature**	300 °C	325 °C	350 °C	300 °C	325 °C	350 °C	300 °C	325 °C	350 °C	300 °C	325 °C	350 °C	300 °C	325 °C	350 °C	300 °C	325 °C	350 °C
**Total CAPEX [€]**	2,992,626	3,211,554	3,456,963	2,986,283	3,202,527	3,378,630	3,013,356	3,211,599	3,285,136	3,001,826	3,214,912	3,299,787	3,005,230	3,168,247	3,363,888	2,962,696	3,162,314	3,453,896
**Total OPEX [€]**	1,228,045	1,229,255	1,256,555	1,228,236	1,229,492	1,243,934	1,228,937	1,230,629	1,241,504	1,229,101	1,230,302	1,240,803	1,228,737	1,229,856	1,243,897	1,227,226	1,231,130	1,239,384
**Total utilities [€]**	52,813	53,713	68,347	53,667	54,619	57,636	53,388	55,113	54,179	53,396	54,450	52,972	53,682	54,986	57,605	54,113	56,285	52,514
**Equipment cost [€]**																		
HEATEX2	125,034	124,943	121,485	121,030	121,303	121,485	125,034	119,938	123,760	122,304	120,029	123,760	123,214	115,934	121,303	66,521	117,117	128,310
COOLER1	13,650	13,741	32,851	13,832	13,923	18,200	13,741	17,927	13,650	13,741	13,923	11,557	13,832	17,836	18,200	13,923	18,291	13,650
PUPM2	244,244	244,244	244,244	236,782	236,782	236,782	245,427	245,427	262,353	253,435	253,435	272,090	237,965	237,965	237,965	237,510	237,510	253,981
CYCLONE	21,749	21,749	21,749	21,749	21,749	21,749	21,749	21,749	21,749	21,749	21,749	21,749	21,749	21,749	21,749	21,749	21,749	21,749
COOLER2	10,283	10,374	12,194	10,374	10,465	10,556	10,374	10,465	10,283	10,374	10,465	9,555	10,374	10,465	10,556	10,374	10,556	9,555
REACTOR2	105,924	108,472	164,346	105,924	108,472	164,346	107,380	108,472	164,346	105,924	108,472	164,346	107,380	108,472	164,346	107,380	108,472	164,346
Sep	20,111	20,111	20,111	20,111	20,111	20,111	20,111	20,111	20,111	20,111	20,111	20,111	20,111	20,111	20,111	20,111	20,111	20,111
HEATER	22,022	15,197	76,713	22,022	15,197	43,680	22,113	15,197	21,749	22,022	15,197	22,204	22,113	15,197	43,680	22,022	15,561	22,113
**Installed cost [€]**																		
HEATEX2	396,214	411,957	410,137	390,936	406,952	410,137	396,214	405,041	413,231	392,665	405,132	411,411	393,848	355,355	408,226	337,428	345,800	663,208
COOLER1	103,922	106,743	163,891	106,834	107,835	112,931	106,652	112,112	103,922	106,743	107,835	104,468	106,834	112,021	112,931	107,835	113,022	103,922
PUPM2	297,206	297,206	297,206	289,653	289,653	289,653	298,389	298,389	315,588	306,488	306,488	325,416	290,836	290,836	290,836	290,381	290,381	307,034
CYCLONE	76,531	76,531	76,531	76,531	76,531	76,531	76,531	76,531	76,531	76,531	76,531	76,531	76,531	76,531	76,531	76,531	76,531	76,531
COOLER2	80,535	82,446	86,632	82,446	83,083	83,265	82,446	83,083	80,535	82,446	83,083	60,879	82,446	83,083	83,265	82,446	83,265	59,969
REACTOR2	316,134	456,456	526,617	316,134	456,456	526,617	329,238	456,456	526,617	316,134	456,456	526,617	329,238	456,456	526,617	329,238	456,456	526,617
Sep	126,581	126,581	126,581	126,581	126,581	126,581	126,581	126,581	126,581	126,581	126,581	132,132	126,581	126,581	126,581	126,581	126,581	126,581
HEATER	132,496	234,052	394,576	132,496	234,052	360,906	132,587	234,052	240,877	132,496	234,052	241,332	132,587	234,052	360,906	132,496	234,416	241,241

Table S4 Heat exchanger and hydrocyclone design for the 10 k t a^{-1} case

Biomass	DSS	DSS	DSS	SS	SS	SS	SM	SM	SM	SMD	SMD	SMD	DBW	DBW	DBW	BW	BW	BW
Temperature	300 °C	325 °C	350 °C	300 °C	325 °C	350 °C	300 °C	325 °C	350 °C	300 °C	325 °C	350 °C	300 °C	325 °C	350 °C	300 °C	325 °C	350 °C
**Heat exchanger**																		
Heat transfer area [m^2]	11.8	11.8	8.2	8.2	8.2	8.2	11.8	7.5	9.7	8.9	7.5	9.7	9.7	3.6	8.2	4.9	1.8	14.7
Number of shells	1	1	1	1	1	1	1	1	1	1	1	1	1	1	1	1	1	1
Front end TEMA symbol	B	B	B	B	B	B	B	B	B	B	B	B	B	B	B	B	B	B
Shell TEMA symbol	E	E	E	E	E	E	E	E	E	E	E	E	E	E	E	E	E	E
Rear end TEMA symbol	M	M	M	M	M	M	M	M	M	M	M	M	M	M	M	M	M	M
Tube material	316LW	316LW	316LW	316LW	316LW	316LW	316LW	316LW	316LW	316LW	316LW	316LW	316LW	316LW	316LW	316LW	316LW	316LW
Tube design gauge pressure [barg]	164	164	164	164	164	164	164	164	164	164	164	164	164	156	164	164	156	164
Tube design temperature [°C]	335	360	375	335	360	375	335	360	390	295	365	385	335	353	365	295	335	390
Tube operating temperature [°C]	264	285	309	260	285	313	276	285	331	260	280	303	260	282	302	260	227	311
Tube outside diameter [m]	0.019	0.019	0.019	0.019	0.019	0.019	0.019	0.019	0.019	0.019	0.019	0.019	0.019	0.019	0.019	0.019	0.019	0.019
Shell material	316L	316L	316L	316L	316L	316L	316L	316L	316L	316L	316L	316L	316L	316L	316L	316L	316L	316L
Shell design gauge pressure [barg]	164	164	164	164	164	164	164	164	164	164	164	164	164	156	164	164	156	164
Shell design temperature [°C]	335	360	375	335	360	375	335	360	390	335	365	385	335	353	365	335	335	390
Shell operating temperature [°C]	300	325	350	299	325	350	300	325	350	300	325	350	300	325	350	300	307	350
Number of tubes per shell	81	81	81	81	81	81	81	81	81	81	81	81	81	81	81	33	81	81
Tube length extended [m]	2.55	2.55	1.80	1.80	1.80	1.80	2.55	1.65	2.10	1.95	1.65	2.10	2.10	1.65	1.80	2.55	1.80	3.15
Tube pitch [m]	0.02	0.02	0.02	0.02	0.02	0.02	0.02	0.02	0.02	0.02	0.02	0.02	0.02	0.02	0.02	0.02	0.02	0.02
Shell diameter [m]	0.26	0.26	0.26	0.26	0.26	0.26	0.26	0.26	0.26	0.26	0.26	0.26	0.26	0.26	0.26	0.18	0.26	0.26
Expansion joint	NO	NO	NO	NO	NO	NO	NO	NO	NO	NO	NO	NO	NO	NO	NO	NO	NO	NO
Number of tube passes	1	1	1	1	1	1	1	1	1	1	1	1	1	1	1	1	1	1
Number of shell passes	1	1	1	1	1	1	1	1	1	1	1	1	1	1	1	1	1	1
**Hydrocyclone**																		
Heat exchanger design tool	E	E	E	E	E	E	E	E	E	E	E	E	E	E	E	E	E	E
Cyclone diameter [meter]	0.5	0.5	0.5	0.5	0.5	0.5	0.5	0.5	0.5	0.5	0.5	0.5	0.5	0.5	0.5	0.5	0.5	0.5
Number of cyclones per manifold	1	1	1	1	1	1	1	1	1	1	1	1	1	1	1	1	1	1
Solid flow rate [kg/hr]	28405	28405	28405	28405	28405	28405	28405	28405	28405	28405	28405	28405	28405	28405	28405	28405	28405	28405
